# Reply to Huth et al.: Cities are defined by their spatially aggregated socioeconomic networks

**DOI:** 10.1073/pnas.2119313118

**Published:** 2022-01-05

**Authors:** Andrew J. Stier, Kathryn E. Schertz, Nak Won Rim, Carlos Cardenas-Iniguez, Benjamin B. Lahey, Luís M. A. Bettencourt, Marc G. Berman

**Affiliations:** ^a^Department of Psychology, University of Chicago, Chicago, IL 60637;; ^b^Department of Public Health Sciences, University of Chicago, Chicago, IL 60637;; ^c^Department of Ecology & Evolution, University of Chicago, Chicago, IL 60637;; ^d^Mansueto Institute for Urban Innovation, University of Chicago, Chicago, IL 60637;; ^e^The University of Chicago Neuroscience Institute, University of Chicago, Chicago, IL 60637

Huth et al. (1) claim that our finding of lower depression rates in larger US cities (2) is “unwarranted.” Their argument is based on an oversight of our fundamental assumption articulating rates of depression to city size: that cities are socioeconomic networks mediated by built environments. Problematically, their analysis is based on a flawed definition of city boundaries. Here, we address each of these points.

First, the issue of functional city definitions: The fundamental insight of urban science (3) is that cities are spatially aggregated socioeconomic networks with properties self-consistently shaped by their built infrastructure. Consequently, meaningful spatial city boundaries must capture—in a single unit of analysis—where people live, socialize, and work. In the United States, the well-tested definition that fits these criteria is the metropolitan statistical area (MSA) as delineated by the US Office of Management and Budget since the 1960s (4) and measured annually since. This is the standard answer to modern cities’ spatially extended socioeconomic networks that include city cores and suburbs and the one we adopt. Thus, though suburbanites may not feel like they live in a city, their interactions are inextricably intertwined with the broader metropolitan area. Similar functional definitions have only recently been proposed for European cities by the Organisation for Economic Co-operation and Development (OECD) (5). Researchers studying US cities must consider MSAs and the long history of their measurement and refinement in the United States.

This brings us to the reanalysis of our data in ref. 1. Huth et al. claim that the scaling of depression rates with city size depends on the spatial definition of cities—defined by them as circles of a given radius around city centers, but provide no methodology for how to define city centers. Because our theory and its predictions for depression rates are mediated by integrated socioeconomic networks, their method, which only captures a fraction of socioeconomic links, cannot be understood with the same models and analytic techniques. Rates of depression obtained this way should vary substantially from the MSA because of movement and sorting of individuals across arbitrary city boundaries.

**Fig. 1.**
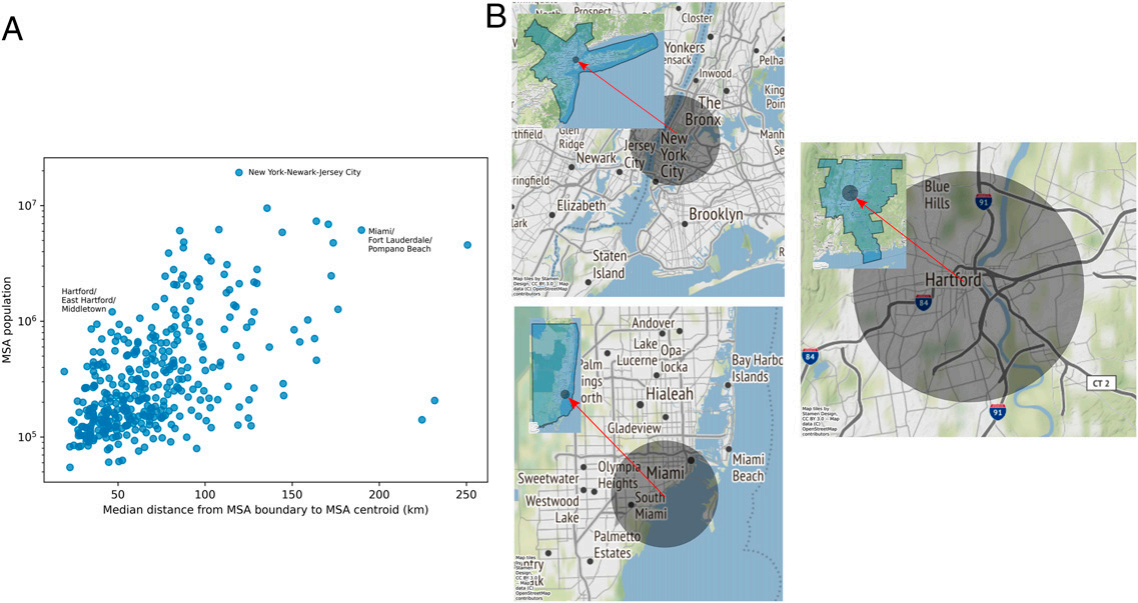
Cities vary substantially in geographic size. (*A*) When defining cities as MSAs, more populated cities tend to also have a greater spatial extent, though this varies considerably with cities’ unique geographies. (*B*) Using distance from city centers to define city boundaries results in incomparable units of analysis across cities, depending on the placement of city centers and the radius used. Displayed city boundaries have radii of 10 km, the smallest radius used in ref. 1. (*Insets*) The extent of the MSA as defined by the US Office of Management and Budget (4).

In addition to this violation of the fundamental assumption of the theory, any new method must deal with large variations in the size and shape of cities (Fig. 1 *A* and *B*, *Insets*). Their method yields incomparable units of analysis as smaller cities will be better contained than larger cities. This is clearly seen in the “city centers” of New York City and Miami, which are not covered by a 10-km radius, but the majority of down town Hartford, CT, is covered (Fig. 1*B*). Thus, this method of defining cities is nonsensical and yields extremely noisy results with wide confidence intervals that result in statistical null results (1). In general, containing “urban cores” would require a variable radius increasing with city population size (Fig. 1*A*), necessitating more expansive city boundaries for larger cities to maintain comparable units of analysis. In summary, defining cities with small distances from unknown city centers isolates incomplete and biased portions of cities' networks (Fig. 1*B*). The analysis in ref. 1 demonstrates a complete disregard for the functional understanding of cities that emerged over the last 60 years.
